# Forecasting medical state transition using machine learning methods

**DOI:** 10.1038/s41598-022-24408-x

**Published:** 2022-11-28

**Authors:** Xiaokai Nie, Xin Zhao

**Affiliations:** 1 School of Automation, Southeast University, Nanjing, 210096 People’s Republic of China; 2 Key Laboratory of Measurement and Control of Complex Systems of Engineering, Ministry of Education, Southeast University, Nanjing, 210096 People’s Republic of China; 3 Shenzhen Research Institute, Southeast University, Shenzhen, 518057 People’s Republic of China; 4 School of Mathematics, Southeast University, Nanjing, 211189 People’s Republic of China

**Keywords:** Computational biology and bioinformatics, Engineering, Mathematics and computing

## Abstract

Early circulatory failure detection is an effective way to reduce medical fatigue and improve state pre-warning ability. Instead of using 0-1 original state, a transformed state is proposed in this research, which reflects how the state is transformed. The performance of the proposed method is compared with the original method under three models, including logistic regression, AdaBoost and XGBoost. The results show that the model XGBoost generally has the best performance measured by AUC, F1 and Sensitivity with values around 0.93, 0.91 and 0.90, at the prediction gaps 5, 10 and 20 separately. Under the model XGBoost, the method with transformed response variable has significantly better performance than that with the original response variable, with the performance metrics being around 1% to 4% higher, and the t values are all significant under the level 0.01. In order to explore the model performance under different baseline information, a subgroup analysis is conducted under sex, age, weight and height. The results demonstrate that sex and age have more significant influence on the model performance especially at the higher gaps than weight and height.

## Introduction

The Intensive Care Unit (ICU) is an organized medical system for critically ill patients, which provides intensive and specialized medical and nursing care, an enhanced capacity for monitoring, and multiple modalities of physiologic organ support to sustain life during a period of life-threatening organ system insufficiency^1^. Real-time state monitoring in ICU supports medical decision by providing massive online data that is instantly processed by clinicians for medical and nursing care actions in most cases. As a frequently used monitoring method, real-time state monitoring makes the physiological signals observed mechanically while they are originally difficult or even impossible to be measured. However, real-time monitoring can only give an alert exactly when the signals are out of range. If clinicians always keep ready for such urgent states, medical fatigue could not be avoided, which will therefore likely lead to serious consequences including low caring efficiency, slow reaction behavior, and medical accidents. Such consequences will have the negative influence on not only the clinicians but also the patients, including slow and non-precision medical treatment, high cost and low surviving rate due to the inaccurate prescription and treatment delay.

State forecasting is an effective approach to reducing such medical fatigue, which works by forecasting the patient state some time ahead using the current monitored signals. If clinicians can be alerted even a few minutes in advance before the urgent state arises, they will have more precious time for the preparation for coping with it, and consequently there will be less intensive requirement for their instant reactions. The medical resources saved can be used for many other purposes. For example, the saved medical expenditure covered by the local governments can be shifted for further medical assistance and thus boost the medical technology development. The improved medical treatment effect can definitely increase the patient survival rate and reduce their medical costs, leading to the remarkable improvement of the overall medical experience.

Due to the advantages of state forecasting, developing suitable specific method becomes essential. The input variables mainly include the physiological signals and drug treatment information, and the response variable is the state of the patient, with 0 representing the current condition that is relatively safe, and 1 indicating that instant medical care is required in response to the occurring of some diseases. The current state forecasting models mainly concentrate on two ways of improving the model performance, which mine more information involved in the input variables, and use better models to explore the relationship between the input and response variables.

The existing methods exploring the information involved in the input variable include: dimension reduction methods to leave out redundant variables, and dimension ascension methods to transform original variables into more variables on multi-resolution levels. Dimension reduction methods mainly include orthogonal-transformation-based principle component analysis, factor analysis and so on. Categorical principal component analysis is used to study the risk factors for healthcare-associated infections in acute cardiac patients^2^. The functional ensemble survival tree is constructed by incorporating multivariate functional principal component analysis to characterize the changing patterns of multiple time-varying neurocognitive biomarker trajectories^3^. Risk factor analysis and nomogram are used for predicting in-hospital mortality in ICU patients with sepsis and lung infection^4^. Dimension ascension methods include resolution decomposition method like wavelet transform. The maximal overlap discrete wavelet transform is used to explore the original variables on different resolution levels^5^. In order to improve the performance of automated detection of sudden cardiac death, the discrete wavelet transform is used to explore the non-stationary characteristics involved in the electrocardiograms signals^6^. These methods aimed to improve the model performance by mining more information contained in the original input variables.

The main models used for describing the relationship between the input and response variables include parametric models like logistic regression, and non-parametric models like decision trees. Parametric models explore the relationship between the input and the response variables by solving the optimum parameters while non-parametric models construct optimum decision rules to predict the best value for the response variable. The logistic regression model is suggested to predict binary outcomes via a mobile application for Android with an example of a real case in ICU^7^. Logistic regression model is applied to explore the relationship between acute kidney injury and in-hospital mortality^8^. The logistic early warning scores is constructed to predict death after cardiac surgery^9^. In addition to logistic regression, parametric models ARIMA, GARCH are also the main contributors for the relationship exploration. The model ARMA is applied to explore the COVID-19 infection process in Italy and Spain^10^. Performance of models ARMA and GARCH is compared with others in the streaming forecasting context^11^. Models based on non-parametric estimation mainly include the machine learning methods like decision trees, ensemble methods like bagging and boosting methods, and neural networks as well as deep learning methods^12^. An interval forecasting model is developed to predict the monitored variables over a few observations ahead^11^. Machine learning models like logistic regression, random forest, and XGBoost are proposed to predict the occurrence of acute kidney injury^13^. A deep learning-driven approach based on a generative adversarial network (GAN) model is developed to predict the length of stay for patients in the ICU^14^.

In fact, one of the difficulties in analyzing such data is caused by their response variable instead of the input variables and their underlying relationship. Compared to the typical survival analysis^15,16^, in which patient keeps staying at state 0 and finally censored at state 1, the state forecasting problem has the state changes between 0 and 1 until the end of the time series. The state transition process renders the forecasting problem complex and challenging. Instead of simply predicting the state  that is either 0 or 1, clinicians focus more on the transition of the state like from 0 to 1or from 1 to 0. If the state stays at 0 or 1, they can maintain their current medical treatment without changing. The response variable with such a switching state can be regarded as a Markov process which describes the transition process among the predefined different states. The current research mainly concentrates on the Hidden Markov model (HMM) and its extensions. For example, HMM and decision trees are combined to estimate the prior distribution for the monitored variables in the ICU^17^, and a coupled HMM is applied to model a sequential contrast patterns based septic shock prediction approach^18^. In addition to the transition property, the class-imbalance phenomenon is also involved in the response variable, which means the proportions of the states are quite imbalanced and thus the forecasting models tend to predict all states as the major class to seek for high accuracy. The disadvantage of such behavior is obvious. With high accuracy, the model may tend to have a good performance in terms of some metrics, but the model becomes meaningless in terms of medical assistance. Instead of using traditional ways to deal with problems caused by class-imbalance phenomenon, the transformed state will replace the original state as the response variable, in which different transition ways can be tested whether they can bring more information than the original states or not. The comparison is conducted under different models including traditional logistic regression and machine learning methods. Beyond the comparison, a subgroup analysis is conducted to compare the model performance under different patient baseline information like age, sex and weight, which are collected instantly at the admission. In this way, the subgroup analysis can help clinicians decide the preferred model as soon as the patient is admitted into the ICU.

In this study, a transition based state forecasting method is proposed to deal with the complex properties involved in the response variable. A subgroup analysis is conducted to compare the performance of the method under different baseline information. The rest of this paper is organized as follows. Section 2 describes the method proposed in this research. Section 3 presents the real medical data analysis. Concluding remarks and perspectives on the further research are given in Section 4. All the computations are implemented using R software^19^.

## Methods

For a specific individual *n*, the response variable is $S_{n,\cdot }$,

$$\begin{aligned} S_{n,\cdot }=\left[ s_{n,1}, s_{n,2}, \cdots , s_{n,T_n} \right] ^T, \end{aligned}$$

in which, $s_{n,t}$ is a random variable changing between 0 and 1, with $n=1,2,\ldots , N$ and $t=1,2,\ldots , T_n$. In order to compare the model performance under the original $S_{n,\cdot }$ and the state transformed response variable, denoted as $S^{*}_{n,\cdot }$, the transition is expressed as follows,

1 $$\begin{aligned} s^{*}_{n,t}=\left\{ \begin{array}{cc} 0&{} s_{n,t}=0 \text { and } s_{n,t+1}-s_{n,t}=0, \\ 1&{} s_{n,t+1}-s_{n,t}=-1, \\ 2&{} s_{n,t+1}-s_{n,t}=1, \\ 3&{} s_{n,t}=1 \text { and } s_{n,t+1}-s_{n,t}=0. \end{array} \right. \end{aligned}$$

In the modeling process, $S_{n,\cdot }$ and $S^{*}_{n,\cdot }$ are the response variables respectively. But afterwards, in order to compare the model performance on the same response variable level, the prediction result denoted as ${\hat{S}}^{*}_{n,\cdot }$ is transformed back to the original state $S_{n,\cdot }$ with values 0 and 1 according to Equation  1. The input monitored variables are the multivariate time series $M_{n,\cdot ,\cdot }$ which is referred to as the matrix containing all monitored variables *K* at all times $T_n$ (the time length for individual *n*) and given below.

$$\begin{aligned} M_{n,\,\cdot ,\,\cdot }=\left[ \begin{array}{cccc} M_{n,1,1} &{} M_{n,2,1} &{} \cdots &{} M_{n,K,1} \\ M_{n,1,2}&{} M_{n,2,2} &{} \cdots &{} M_{n,K,2} \\ \vdots &{} \ddots &{} \vdots \\ M_{n,1,T_n} &{}M_{n,2,T_n} &{} \cdots &{} M_{n,K,T_n} \end{array} \right]. \end{aligned}$$

If the response variable is forecasted over *gap* observations ahead, then the model established for the individual *n*  is given by

$$\begin{aligned} {\hat{S}}_{n,\, gap+1:T_n}= & {} {\hat{f}}(M_{n,\,\cdot ,\,1:T_n-gap}), \\ {\hat{S}}^{*}_{n,\,gap+1:T_n}= & {} \hat{f^*}(M_{n,\,\cdot ,\,1:T_n-gap}). \end{aligned}$$

The model *f*(.) has various choices including logistic regression, AdaBoost and XGBoost. Following the modeling process, ${\hat{S}}^{*}_{n,\cdot }$ is transformed back to ${\hat{S}}_{n,\cdot }$ for comparison.

### Models for classification

Logistic regression belongs to the generalized linear regression, which is an umbrella term that encompasses many other models, allowing the response variable to have an error distribution other than a normal distribution. Instead of using the original response variable, logistic regression has the function of response as the new variable, like Normal, Poisson, and binomial responses. The link function chosen for logistic regression is binomial. For multinomial logistic regression, also referred to as the Softmax model, the formula is given by

$$\begin{aligned} P(S=k|M)=\frac{e^{w_kM}}{1+\sum _{k=1}^{K}e^{w_kM}}, \end{aligned}$$

where $w_k$ is the parameter vector for the category *k*. The parameter estimation method is maximal likelihood estimation and the predicted category is the one with highest probability.

AdaBoost, namely Adaptive Boosting, is an ensemble method working by having higher weights assigned to incorrectly classified instances at each iteration to improve the performance. AdaBoost is an additive model using forward stagewise algorithm with the exponential loss function $L(s,{\hat{s}})=e^{(-s{\hat{s}})}$. Based on the loss function, the weight for the model $G_r(m)$ and the weighted misclassification rate at iteration *r* are

$$\begin{aligned} \alpha _r=\frac{1}{2}log\frac{1-e_r}{e_r},\,\,e_r=\sum _{s_t\ne {\hat{s}}_t}\omega _{r,t}, \end{aligned}$$

where $\alpha _r$ decreases as $e_r$ increases. The updating method for $\omega _{r,t}$ is

$$\begin{aligned} \omega _{r+1,t}=\frac{\omega _{r,t}}{Z_r}e^{-\alpha _rs_tG_r(m_t)},\,\, Z_r=\sum _{t}\omega _{r,t}e^{-\alpha _rs_tG_r(m_t)}. \end{aligned}$$

The final AdaBoost model is defined as

$$\begin{aligned} G(m)=sign\{\sum _{r}\alpha _rG_r(m)\}. \end{aligned}$$

The basic model $G_r(m)$ with better performance are assigned with higher weight. If the value $\sum _{r}\alpha _rG_r(m)$ is positive, then *G*(*m*) is 1, and vice verse.

XGBoost, short for eXtreme Gradient Boosting, is also an additive model composed of *m* basic models,

$$\begin{aligned} {\hat{s}}_t^{(r)}={\hat{s}}_t^{(r-1)}+g_r(m_t). \end{aligned}$$

Compared with AdaBoost, the optimization function $L_c$ is a combination of loss function $L(s,{\hat{s}})$ and regularization term $\Omega (g_r)$ to control the model complexity,

$$\begin{aligned} L_c=\sum _{t}L(s_t,{\hat{s}}_t)+\sum _{r}\Omega (g_r). \end{aligned}$$

The advantage of XGBoost is that it approximates its optimization function $L_c$ by the second order Taylor expansion, which gained better performance than the first order model Gradient Boosting Decision Tree (GBDT). The second order Taylor expansion of $L_c$ is expressed as

$$\begin{aligned} L_c^r&=\sum _{t}L(s_t,{\hat{s}}_t^{(r-1)}+g_r(m_t))+\sum _{i\le r}\Omega (g_r)\\&\quad =\sum _{t}[L(s_t,{\hat{s}}_t^{(r-1)}+\frac{\partial L(s_t,{\hat{s}}_t^{(r-1)})}{\partial {\hat{s}}_t^{(r-1)}}g_r(m_t)+\frac{\partial ^2 L(s_t,{\hat{s}}_t^{(r-1)})}{\partial ({\hat{s}}_t^{(r-1)})^2}g^2_r(m_t))]+\Omega (g_r)+\sum _{i\le r-1}\Omega (g_r)\\&\quad \simeq \sum _{t}[\frac{\partial L(s_t,{\hat{s}}_t^{(r-1)})}{\partial {\hat{s}}_t^{(r-1)}}g_r(m_t)+\frac{1}{2}(\frac{\partial ^2 L(s_t,{\hat{s}}_t^{(r-1)})}{\partial ({\hat{s}}_t^{(r-1)})^2}g^2_r(m_t))]+\Omega (g_r). \end{aligned}$$

If the basic model is decision tree, by assuming that the sample $m_t$ falls into the terminal node *j*, the corresponding indicator variable can be represented as ${\textbf{1}}_j=\{i|q(m_t)=j\}$, where ${\textbf{1}}$ is the indicator variable with value as 1 if the condition are satisfied. The score for terminal node *j* is defined as $\omega _j$. The total number of terminal nodes is *T*. The regularization term is defined as

$$\begin{aligned} \Omega (g_r)=\gamma T+\frac{1}{2}\lambda \sum _{j=1}^T \omega _j^2, \end{aligned}$$

which balances the complexity of the tree defined by the number of terminal nodes and the score value for each node. $\gamma$ and $\lambda$ are balanced weights which can be optimized using cross validation. In this way, the basic function $g_r(m_t)$ becomes $\omega _{q(m_t)}$ and the optimization function $L_c$ becomes

$$\begin{aligned} L_c^r\simeq \sum _{j=1}^{T}[\sum _{i\in {\textbf{1}}_j} \frac{\partial L(s_t,{\hat{s}}_t^{(r-1)})}{\partial {\hat{s}}_t^{(r-1)}}\omega _j+\frac{1}{2}(\sum _{i\in {\textbf{1}}_j} \frac{\partial ^2 L(s_t,{\hat{s}}_t^{(r-1)})}{\partial ({\hat{s}}_t^{(r-1)})^2}+\lambda )\omega _j^2)]+\gamma T. \end{aligned}$$

It follows that the best value of parameter $\omega _j$ at iteration *r* is obtained as

$$\begin{aligned} \omega ^*_j=\mathop {\arg \min }\limits _{\omega _j} L_c^r. \end{aligned}$$

The structure of the tree can be found using greedy algorithm or approximation algorithm by choosing the best split which maximizes the $L_c$ gain.

### Performance metrics and subgroup analysis

The performance under the original variable and the transformed variable are compared using AUC, F1 and Sensitivity. The metric Sensitivity describes the rate of diagnosed positive (predicted state as 1) out of true positive (real state as 1), which is intensively cared by the clinicians. The comprehensive metric of Specificity and Sensitivity used is the AUC value, which balances the accuracy of both positive rate and negative rate. The metrics F1 is also a comprehensive performance metric. A model with higher values in these metrics (maximum as 1) has the better performance than others. The definitions of these metrics are as follows. The number of True Positive for individual *n* is denoted as $TP_n$, and the others are similarly defined. Let $f(i,j)=\sum _{t=gap+1}^{T_n}{\textbf{1}}\{s_{n,t}=i \wedge {\hat{s}}_{n,t}=j\}$, $i,j\in \{0,1\}$, then

$$\begin{aligned}{}[TP_n,\,FP_n,\,FN_n,\,TN_n]=[f(1,1),\,f(0,1),\,f(1,0),\,f(0,0)]. \end{aligned}$$

The Sensitivity and Specificity are defined as

$$\begin{aligned} se_n=\frac{TP_n}{TP_n+FN_n}, \,\,sp_n=\frac{TN_n}{FP_n+TN_n}. \end{aligned}$$

The precision and F1 are defined as

$$\begin{aligned} pre_n=\frac{TP_n}{TP_n+FP_n}, \,\, F1_n=\frac{2pre_n*se_n}{pre_n+se_n}. \end{aligned}$$

The AUC value is defined as the area under the ROC curve with 1-Specificity as the x lab, and Sensitivity as the y lab. For each individual *n*, the model is trained with around $70\%$ of data and the rest data are used for testing. The performance metrics are all computed from the test data. To test whether there is a significant difference between the two methods (the original forecasting and the transformed forecasting), t test is conducted to analyze their performance under different forecasting gaps and machine learning models.

In order to explore whether the model performance varies among different individuals or not, a subgroup analysis is conducted by using model ANOVA to test whether or not the baseline information has significant influence on the model performance. In the model, the response variable is

$$\begin{aligned}{}[ AUC_.,\, F1_.\,se_.], \end{aligned}$$

and the baseline variables $Z_{n,\cdot }$, such as age, sex and weight across the individuals are the input variables:

$$\begin{aligned} \left[ \begin{array}{cccc} Z_{1,1}&{}Z_{1,2}&{}\cdots &{}Z_{1,N_Z}\\ Z_{2,1}&{}Z_{2,2}&{}\cdots &{}Z_{2,N_Z}\\ \cdots &{} \cdots &{}\ddots &{}\cdots \\ Z_{N,1}&{}Z_{N,2}&{}\cdots &{}Z_{N,N_Z} \end{array} \right] . \end{aligned}$$

It follows that the subgroup model can be given by

$$\begin{aligned}{}[ AUC_.,\, F1_.\,se_.]=g\{Z_{1},\,Z_{2},\,\cdots ,\,Z_{N_Z}\}. \end{aligned}$$

Consequently, from the model *g*, whether the baseline variables have significant influence on the model performance or not can be quantitatively measured and identified.

## Real data analysis

This dataset origins from HiRID^20^, which is a freely accessible critical care dataset containing the data relating to almost 34 thousands patient admissions to the Department of Intensive Care Medicine of the Bern University Hospital, Switzerland (ICU). The original dataset has been imputed and processed by the research^20^, with 18 input variables left, including bedside monitored variables like heart rate, lab test variables like serum glucose, and drug presence variable indicating whether the drug is used or not. The response variable is an indicator variable, with value as 1 if circulatory failure occurs and value as 0 if not. The dataset is down sampled to a five-minute time grid with missing value imputed. After deleting datasets with only one state either in training data ($70\%$) or the whole data, the individuals left in this research is 18210. As the whole dataset is quite large, the description of the dataset is based on the randomly selected 70 individuals with 123160 observations in total.

**Figure 1:**
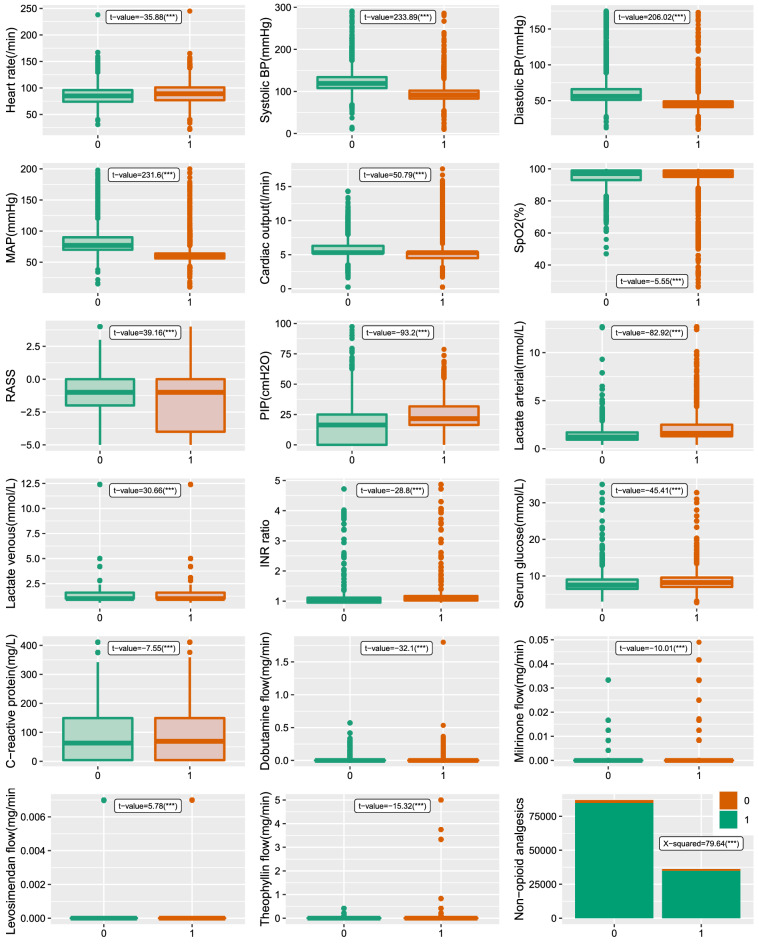
The variables information under state 0 and 1. The state 0 means the individual is not in circulatory failure and 1 means circulatory failure which should be alerted. The t value is based on the student t-test, and chi-squared test is conducted for the categorical variable Non-opioid analgesics, with all the p-value significant under level 0.01(***).

From Fig. 1, it is clear that all the 18 input variables have significant relationships with the variable state. For example, individuals under circulatory failure generally have lower MAP, Cardiac output, RASS and so on. The general standard for MAP is 65 mmHg, and a value lower than that indicates a higher death rate. Vasopressors drugs should be applied under lower MAP. For variable RASS, it has wider range and lower value under state 1. The Richmond Agitation Sedation Scale (RASS) is an instrument designed to assess the level of alertness and agitated behavior in critically-ill patients. It demonstrates the fluctuating levels of consciousness. Lower RASS represents lower consciousness, and individuals are more likely to be at state 1. The distributions of the variable RASS under state 0 and 1 are also different. Variable Systolic blood pressure (BP) indicates how much pressure the blood is exerting against the artery walls when the heart beats pumping blood out. A lower Systolic BP indicates a higher possibility at state 1 according to the Fig. 1. The variable diastolic blood pressure (BP) shows similar behavior like that of Systolic BP. Cardiac output is an important metric reflecting the Cardiac dysfunction, which is an important consequence of circulatory failure that affects mortality. For variables like PIP, Lactate arterial, they generally have the higher values under the state circulatory failure. The peak inspiratory pressure (PIP) is the highest pressure measured during the respiratory cycle and is a function of both the resistance of the airways and the compliance of the respiratory system. High PIP is associated with pneumothorax and reduction in cardiac output, which indicates a possibility of circulatory failure. Lactate arterial is highly related with serum lactate level. When MAP is lower than 65 mmHg and serum lactate level is higher than 2 mmol/L, the in-hospital mortality rate can be over 40%. Overall, all the input 18 variables have significant relationships with the response state.

**Table 1:** Performance results for different forecasting gaps and methods with the original response variable and the transformed response variable. The gaps have values 1, 5, 10 and 20. Under each performance metric, four values are colored representing the best performance under the four gaps. The red one means that the transformed response variable has better performance, while the blue one indicates the original variable is better.

Performance	Model	Logistic regression				AdaBoost				XGBoost			
	gap	1	5	10	20	1	5	10	20	1	5	10	20
AUC	Ori mean	0.869	0.850	0.803	0.777	0.933	0.925	0.902	0.899	0.933	0.926	0.908	0.906
	Ori sd	0.087	0.094	0.115	0.126	0.056	0.060	0.070	0.073	0.056	0.060	0.069	0.070
	Tra mean	0.867	0.844	0.800	0.779	0.919	0.919	0.899	0.914	0.929	0.933	0.920	0.936
	Tra sd	0.085	0.095	0.115	0.126	0.060	0.069	0.076	0.078	0.057	0.062	0.063	0.064
	t-value	2.704	6.635	2.541	-1.231	23.58	9.396	3.282	-18.90	6.909	-11.06	-17.59	-42.98
	p(t)	0.007	0.000	0.011	0.218	0.000	0.000	0.001	0.000	0.000	0.000	0.000	0.000
F1	Ori mean	0.822	0.798	0.733	0.694	0.907	0.899	0.868	0.864	0.908	0.900	0.877	0.875
	Ori sd	0.139	0.153	0.200	0.229	0.084	0.089	0.109	0.112	0.086	0.090	0.109	0.108
	Tra mean	0.816	0.786	0.726	0.695	0.887	0.892	0.868	0.890	0.901	0.910	0.894	0.917
	Tra sd	0.140	0.159	0.202	0.227	0.093	0.102	0.113	0.112	0.088	0.093	0.097	0.092
	t-value	4.078	7.303	3.124	-0.467	21.61	7.036	-0.276	-21.97	7.321	-9.566	-15.60	-40.21
	p(t)	0.000	0.000	0.002	0.641	0.000	0.000	0.783	0.000	0.000	0.000	0.000	0.000
Sensitivity	Ori mean	0.819	0.791	0.715	0.673	0.911	0.898	0.860	0.856	0.909	0.895	0.866	0.863
	Ori sd	0.166	0.183	0.235	0.265	0.098	0.105	0.130	0.136	0.102	0.110	0.133	0.133
	Tra mean	0.816	0.781	0.712	0.677	0.891	0.888	0.856	0.877	0.903	0.905	0.884	0.908
	Tra sd	0.164	0.185	0.236	0.263	0.109	0.120	0.140	0.139	0.104	0.109	0.120	0.114
	t-value	1.662	4.938	1.560	-1.361	18.49	8.428	2.827	-15.06	5.842	-8.737	-14.00	-34.14
	p(t)	0.097	0.000	0.119	0.174	0.000	0.000	0.005	0.000	0.000	0.000	0.000	0.000

The models used in the process include Logistic regression, AdaBoost and XGBoost. The prediction gap has values 1, 5, 10 and 20. The response variables include the original state and the transformed state. From Table 1, averaged from all the individuals, all the methods have the performance increases as the gap decreases. Models tend to have better performance when the prediction gap is small. But, the best model XGBoost among the three models has the best performance, even when the gap becomes 10 or 20, with values over or around 0.9 across AUC, F1 and Sensitivity. In terms of gap 1, XGBoost has similar performance to that of AdaBoost, being 0.933 in AUC with standard deviation 0.056, around 0.9 in F1 and Sensitivity with standard deviation 0.08 and 0.1. When the gap is 5, 10 or 20, XGBoost has generally better performance than the others in both the mean value and the standard deviation, mostly being around 0.93 in AUC, 0.91 in F1, and 0.9 in Sensitivity.

Through comparing the method using the transformed state with that using the original state, it is found that they share similar performance for Logistic regression and AdaBoost. But when it comes to the model XGBoost, the transformed states have significantly better performance than the original one, generally having values of 0.01 higher in all three metrics. The corresponding standard deviation is similar to or smaller than that of the original one especially when the gap is 20. A higher value in AUC or F1 represents a better overall performance, while a higher value in sensitivity represents a higher ability in detecting the circulatory state, which is especially important for the clinicians. It can be seen that the XGBoost model with the transformed states achieved more satisfactory performance across the possible gaps.

**Figure 2:**
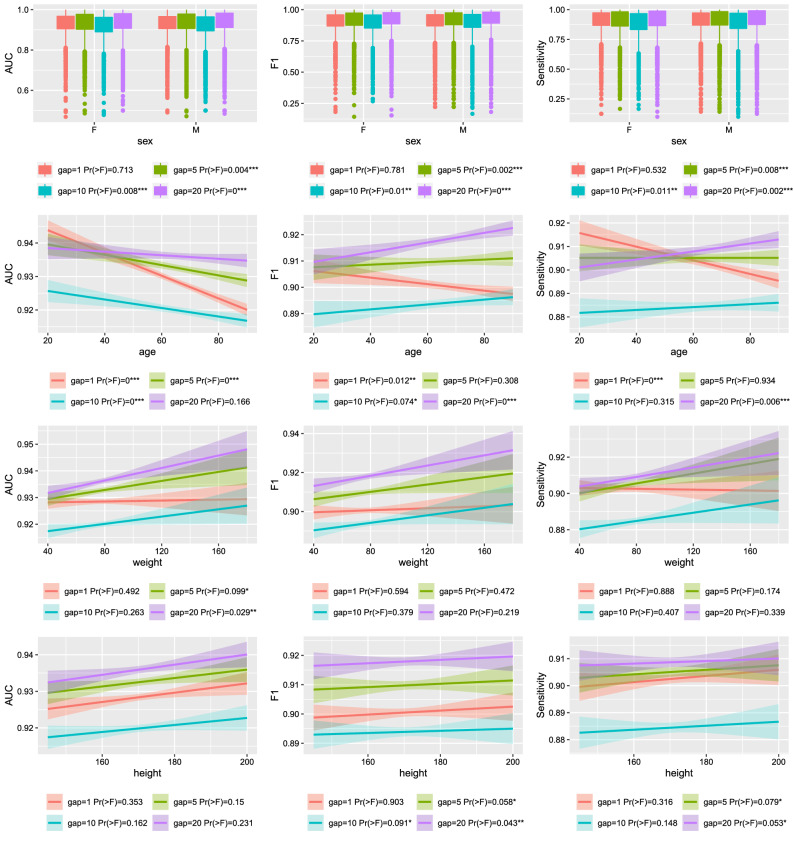
The results of ANOVA test between XGBoost performance and the baseline information under different prediction gaps. The 95% Confidence Interval of linear regression is shown as the shadow area. The probability of the F value is resulted from the ANOVA test. The corresponding significant levels include: 0.001(***), 0.01(**), 0.1(*).

Figure 2 shows the results of the XGBoost model performance under different baseline information. For the baseline variable sex, the model performance metrics AUC, F1 and Sensitivity all have significantly different values under gaps 5, 10 and 20. Except gap 1 (not significant), males tend to have a little bit higher performance than that of females under gaps 5, 10 and 20, being 0.928 (0.928), 0.934 (0.931), 0.921 (0.918), 0.937 (0.933) for males (females) in AUC, being 0.901 (0.900), 0.911 (0.907), 0.895 (0.891), 0.920 (0.914) for males (females) in F1, and being 0.902 (0.903), 0.907 (0.902), 0.886 (0.881), 0.911 (0.905) for males (females) in Sensitivity. In terms of the baseline variable age, AUC decreases significantly as age increases for all the gaps, F1 increases for gap 10 and gap 20 but decreases under gap 1, and Sensitivity decreases under gap 1 but increases under gap 20. In terms of the baseline variable weight, all performance metrics increase but only significantly under gap 5 and 20 for variable AUC. In terms of the baseline variable height, the performance metrics increase but only significant under gaps 5 and 20 for Sensitivity.

## Conclusion

Aiming to forecast the circulatory failure status, this study develops the methods based on the models Logistic regression, AdaBoost and XGBoost. The highlight of this study is that, instead of the original state, the transformed state representing the way in which the states are transformed from the previous states is used as the response variable. The transformed states contain more information than the original states. In order to compare their performance on the same level, the predicted transformed states are transformed back to states 0 and 1. The results demonstrate that, XGBoost has the best performance among all the models especially at gaps 5,10 and 20. XGBoost and AdaBoost share the similar performance at gap 1. Among the XGBoost results, methods based on transformed response variables have significant better performance than that of the original variables among all the performance metrics AUC, F1, and Sensitivity. A better performance in Sensitivity means the circulatory failure state has the higher chance to be detected, which is of critical importance for clinicians.

In order to investigate whether different individuals have the same good performance or not, the performance of XGBoost is further compared under different baseline information, including age, sex, weight and height. The model ANOVA is applied to test the significance among the performance metrics AUC, F1, Sensitivity and the baseline variables. The results show that sex has some extent of influence to AUC, F1 and Sensitivity, especially at higher gaps. Age has lower but still significant influence on the model performance. The baseline variables weight and height have some significant values indicating dataset with higher weight and height has higher performance. This subgroup analysis gives a method to explore how the baseline information influences the model performance.

In the further research, a prior value for the model parameters can be given based on the subgroup analysis for parametric models. For non-parametric models, a suggested model can be given based on the baseline information. A good prior information can facilitate improving the performance of forecasting especially at the beginning of the modeling compared to randomly assignment. In terms of the input variables, more information can be included in the further research, like medical image data, gene data and pharmacy information. Research on these variables is multimodal data analysis, which combines data information on different levels. Different kinds of multimodal inputs require complex feature extraction and combination methods. The multimodal variables are also collected at different times, which means the information combination is not only at the feature level but also at the time level. Developing such a dynamic model of combining the features that sequentially arise is expected to be a challenging but valuable research direction.

## Data Availability

The source code in the method are available from the corresponding author upon request. The real data in the application can be requested from the reference^21^.
